# Environmental Profile of a Community’s Health (EPOCH): An Ecometric Assessment of Measures of the Community Environment Based on Individual Perception

**DOI:** 10.1371/journal.pone.0044410

**Published:** 2012-09-04

**Authors:** Daniel J. Corsi, S. V. Subramanian, Martin McKee, Wei Li, Sumathi Swaminathan, Patricio Lopez-Jaramillo, Alvaro Avezum, Scott A. Lear, Gilles Dagenais, Sumathy Rangarajan, Koon Teo, Salim Yusuf, Clara K. Chow

**Affiliations:** 1 Population Health Research Institute, McMaster University, Hamilton, Ontario, Canada; 2 Department of Society, Human Development and Health, Harvard School of Public Health, Boston, Massachusetts, United States of America; 3 European Centre on Health of Societies in Transition, London School of Hygiene and Tropical Medicine, London, United Kingdom; 4 Cardiovascular Institute & Fuwai Hospital, Chinese Academy of Medical Sciences, Beijing, China; 5 St. John’s Research Institute, Bangalore, Karnataka, India; 6 Research Direction, Fundacion Oftalmologica de Santander-Clinica Carlos Arila Lulle and Medical School, Universidad de Santander, Bucaramanga, Colombia; 7 Dante Pazzanese Institute of Cardiology, São Paulo, Brazil; 8 Faculty of Health Sciences, Simon Fraser University, Vancouver, British Columbia, Canada; 9 Institut Universitaire de Cardiologie et de Pneumologie de Québec, Université Laval, Québec City, Québec, Canada; 10 The George Institute for Global Health and Sydney Medical School, University of Sydney, Sydney, Australia; University of Hong Kong, China

## Abstract

**Background:**

Public health research has turned towards examining upstream, community-level determinants of cardiovascular disease risk factors. Objective measures of the environment, such as those derived from direct observation, and perception-based measures by residents have both been associated with health behaviours. However, current methods are generally limited to objective measures, often derived from administrative data, and few instruments have been evaluated for use in rural areas or in low-income countries. We evaluate the reliability of a quantitative tool designed to capture perceptions of community tobacco, nutrition, and social environments obtained from interviews with residents in communities in 5 countries.

**Methodology/ Principal Findings:**

Thirteen measures of the community environment were developed from responses to questionnaire items from 2,360 individuals residing in 84 urban and rural communities in 5 countries (China, India, Brazil, Colombia, and Canada) in the Environmental Profile of a Community’s Health (EPOCH) study. Reliability and other properties of the community-level measures were assessed using multilevel models. High reliability (>0.80) was demonstrated for all community-level measures at the mean number of survey respondents per community (n = 28 respondents). Questionnaire items included in each scale were found to represent a common latent factor at the community level in multilevel factor analysis models.

**Conclusions/ Significance:**

Reliable measures which represent aspects of communities potentially related to cardiovascular disease (CVD)/risk factors can be obtained using feasible sample sizes. The EPOCH instrument is suitable for use in different settings to explore upstream determinants of CVD/risk factors.

## Introduction

Place of residence is an important determinant of health [1]; yet the empirical evidence describing the diverse mechanisms involved remains limited. We have previously described a conceptual framework which identified multiple social, legislative, and physical domains within the community environment that may have the potential to influence health behaviours and cardiovascular risk factors (e.g. smoking, diet, and physical activity) within populations. [2] The literature we reviewed to generate that model identified the importance of capturing both objective, observable aspects of the environment and how it is perceived by those living in it. For example, smoking prevalence in a community may be shaped both by anti-smoking legislation established in workplaces or other public areas [3]–[4] and by what is socially acceptable. [5].

However, existing methods to measure and quantify environments based on their ability to influence behaviours such as smoking have typically used one or other, and have been limited to a single domain (e.g. policy). Consequently, they fail to capture completely the multiple pathways through which these influences may occur. We therefore developed a novel instrument, the Environmental Profile of a Community’s Health (EPOCH) that could be used to simultaneously collect data on a range of environmental characteristics potentially associated with cardiovascular risk factors and which combined objective measures and perceptions of the environment. [6].

The EPOCH instrument has built on existing measurement approaches from several disciplines, including the physical activity[7]–[8] and sociological [9] literatures. As noted above, strategies for measuring environmental settings fit broadly into two categories; 1) the systematic description of communities, by means of either structured observations or municipal census and/or geographic data, and 2) collection of perception-based measures obtained through interviews with community residents.[9]–[11] While each approach has certain advantages and they can be complimentary [2], methodological challenges exist when attempting to integrate across the strategies. Specifically, a mismatch can occur between data collected through systematic observation of communities, which takes place at the group level, and data collected from survey respondents which occurs at the individual level. In order to appropriately integrate and evaluate ecological data in EPOCH collected from multiple sources, we have adopted a multilevel framework, termed “ecometrics”, which appropriately accounts for the different levels of data collection. [9], [12]–[13].

In the EPOCH instrument, we have incorporated the complimentary strategies of structured observation of communities (EPOCH 1) with a survey of community residents (EPOCH 2). In a previous paper we reported good reliability in the community-level observations obtained in EPOCH 1 in a diverse sample of communities in five countries. [6] In the present study, we further evaluate the ecometric properties (including interrater reliability) of the EPOCH 2 component of the instrument in terms of capturing perceptions of the community tobacco, nutrition, and social environments derived from an interview-based survey of residents from urban and rural communities in five countries.

## Methods

### Research Design

Within the context of the “PURE” study, an international cohort study collecting data on subjects in urban and rural areas in countries worldwide at different levels of development [14], we developed a novel instrument, the Environmental Profile of a Community’s Health (EPOCH), to evaluate communities in terms of multiple environmental factors with potential relevance to risk factors for cardiovascular disease (CVD). [6], [14] The overall design of EPOCH was based on a systematic review of the relationship between environmental factors and CVD and, in particular, the existing instruments used to capture features of the environment. [2] This paper deals with the second element, **EPOCH 2**.

Data are from the initial phase of the EPOCH project, conducted in 84 urban and rural communities from several regions in five countries. [6] The countries and regions involved were China (Yunnan, Qinghai, Beijing, Jiangsu, Shandong, Shanxi, Shannxi, Jiangxi, Liaoning, Xinjiang, Sichuan provinces), India (Karnataka state), Colombia (Santander, Nariño, Quindio, Bolivar), Brazil (Sao Paulo, Angatuba, North region) and Canada (British Colombia, Ontario and Quebec). Communities are a sub-set of the PURE study, which now includes 626 communities in 17 countries, and has recruited over 390,000 subjects, of whom 150,000 subjects are between the ages of 35 and 70 years and among whom extensive information on CVD risk factors has been collected. [15].

Communities were defined as groups of individuals sharing common characteristics and residing in a defined geographic area. [14] They have been designated by local country investigators to align with administrative borders (such as census tracts or postal zones). Rural communities in India, China or Colombia were defined by village boundaries. In urban areas, selected urban communities in each country were sampled across different local income strata to capture diversity of conditions within urban areas. Communities in PURE were selected based on feasibility for long-term follow-up and to maximize the variation in social, physical, and environmental factors within and between countries.

In PURE, the sampling of individuals was carried out within communities with the objective to achieve a representative sample of adults aged 35 to 70 years who intended to continue living in that community for at least 4 years. [14] All eligible individuals in selected communities were invited (either by telephone contact or household visits) to participate in PURE. The EPOCH study was done in a subset of communities in PURE. The EPOCH 1 environmental profile report was obtained from 93 communities, however for logistic reasons EPOCH 2 interviews were only conducted in 84 communities. Sampling of individuals for EPOCH 2 was carried out by approaching a convenience sample of PURE participants from the 84 communities with the aim to recruit 30 individuals (or all participants in smaller communities) with equal numbers of men and women per community. In total, 2,381 completed interviews were obtained with an overall response rate of 92%. The EPOCH instruments were approved by the Hamilton Health Sciences/McMaster Health Sciences Research Ethics board. Written informed consent was obtained from all participants in the study.

### Measures

EPOCH 2 is a structured interview covering three domains relevant to cardiovascular risk factors: 1. the community tobacco environment; 2. the community nutrition environment; and 3. the community social environment. [2] Questions on other CVD risk factors including alcohol and physical activity were not available in this version of the EPOCH 2 questionnaire, but were covered in the complimentary EPOCH 1 component of the instrument. [6] A copy of the version of the EPOCH 2 questionnaire used in this study is provided in **Appendix S1**.

We developed a series of thirteen scales to measure different dimensions of the community environment within each of the three domains of EPOCH 2. Scales were constructed from individual questionnaire items, grouped according to theoretical constructs identified in our literature review and guided by an exploratory factor analysis to empirically confirm that items were measuring a common latent factor.

The community tobacco environment was represented by 2 scales derived from 5 questions with Likert-type responses and 5 scales based on yes/no responses to between 3 and 8 questions. Subjects were asked about the current restrictions on smoking in their communities and their preferences for smoking in public places. Respondents were also asked whether they had seen advertisements both for and against smoking in media, to give their opinion of the social acceptability of smoking, and about their knowledge of the health effects of smoking. The community nutrition environment was measured using 5 scales based on between 2 and 9 yes/no responses to questions on whether residents had seen junk food and/or fruit and vegetable advertisements in media, their awareness of dietary health promotion, their knowledge of dietary causes of CVD, and their awareness of food policy legislation. The community social environment was captured through a single scale with 2 items conceptually related to social cohesion, trust and health behaviors. Respondents were asked if they agreed that “adults in this community tell children, who are not their own children, to stop smoking,” and “people generally help others not related to them in this community”.

### Statistical Analysis

We aggregated across residents from the same community for each of the community-level measures of interest. The community-level reliability of each scale depends on the item consistency and number of items (which contribute to perceptions and measurement error at the individual level), the number of respondents per community, and the degree of intersubjective agreement within communities (measurement error at the community level). The measurement of community-level environmental characteristics based on individual responses is inherently multilevel; to account for this we followed the ecometric methods described by Sampson and Raudenbush.[12]–[13].

Our data had the following four-level structure. The first (lowest) level of variation is represented by items nested within respondents, with the sample size equal to scale items (varying between 2 and 9). The second level is persons nested within communities, and the sample size is the number of respondents (n = 2,381). The third and fourth levels of the hierarchy are communities and countries, with the samples sizes at these levels being the number of communities (n = 84) and countries (n = 5), respectively.

We calibrated four-level linear (for scales with Likert-type items) and logistic (for scales with yes/no items) multilevel models with item responses *i*, within individual *j* in community *k* and country *l* as the outcome,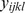. The full linear multilevel random intercepts model is given as:


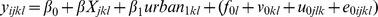
(1) [16]

Within individuals, responses depend on the “true perception” (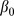) and a random effect attributed to between-item inconsistency (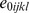). [12] At the second level, perceptions were allowed to vary between individuals as a function of the “true” value of the community scale (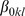), individual demographic characteristics (age, gender, smoking status, and education) (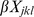), and between-individual differences (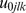). At the third level of analysis, we allowed for a community-specific characteristic (urban or rural, 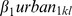) in the fixed part of the model, and at the third and fourth levels of analysis, we allowed for between-community (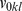) and between-country (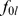) variation in individual responses to scale items.

This analytic approach allowed for the estimation of the following properties for each community-level measure: the item inconsistency (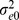), the between-individual differentials in agreement (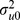), and the between-community (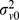) and between-country variation (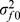). Based on these variance parameters, we estimated the reliability of community-level measures using the following formula [12]:
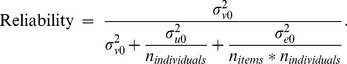
(2)


We calculated the contribution of the between-community variation to the total variance in each scale using the variance partitioning coefficient (VPC). [16].

At the community level, the VPC (also called the intraclass correlation) is a measure of similarity in the responses across questionnaire items between two randomly chosen individuals from the same community. The VPC can vary between 0 and 1 with higher values indicating greater similarity in responses between individuals from the same community and is defined as:
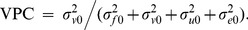
(3)


Following reliability analyses, we estimated multilevel confirmatory factor analysis (CFA) models for each of the scales to explore the extent to which responses to questionnaire items are represented by a common factor at the community level. [17] In addition, we estimated correlations between scales to at the community level to explore interdependence between scales at this level.

Multilevel models were estimated using Stata (version 11) and *MLwiN* (version 2.25).[18]–[19] Multilevel models in our analysis were estimated using Markov chain Monte Carlo (MCMC) simulation and the Metropolis-Hastings algorithm available in *MLwiN*. [17] MCMC procedures were used to reduce bias in the estimates of the random effects parameters, which can arise when fitting multilevel models with binary outcomes using maximum-likelihood procedures. [20] We conducted the analysis in two stages. First, the overall sample was analyzed, and second, the analyses were repeated stratifying the sample according to urban or rural location.

In addition, although our use of MCMC methods ensures that the variance estimates at higher levels are not downwardly biased, the few number of countries (n = 5) means that the standard errors for variance estimates at this level may be large. To account for this, we conducted a sensitivity analysis where countries were treated as ‘fixed’ covariates instead of as a separate level in the multilevel model. We then re-estimated the community-level VPC using Equation 3 without the term for country-level variance (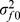).

## Results

A total of 2,381 respondents participated in EPOCH 2 interviews, with an average of 28 respondents across the 84 communities (min/max: 1/60). Twenty-one (<1%) individuals were missing data on one or more of the following covariates, age, gender, smoking status (defined as a current, former, or never smoker), or education (no education, primary school, high school, trade school, or college/university) and were excluded, yielding a final analytic sample of 2,360 individuals in 84 communities and 5 countries. Sample characteristics of the EPOCH 2 participants are presented in **Table 1**. EPOCH 2 respondents were typically aged 53 years, 51% were women, 32% had a high school education, and 29% were current smokers. The EPOCH 2 respondent profile was slightly older, and had more males and current smokers compared to the overall PURE population where the average age was 50 years, 58% were female, 38% had a high school education, and 21% were current smokers. [15].

**Table 1:** Sample characteristics from the EPOCH 2 study in 5 countries.

**Countries (n)**	5
Number of communities *M* (min/max)	16.8 (6/30)
**Communities (n)**	84
Number of individuals *M* (min/max)	28.1 (1/60)
**Individuals (n)**	2381
Age *M* (SD)	53.0 (10.0)
Female (%)	51.4
Education (%)	
No education	12.2
Primary	23.0
Secondary/high school	32.0
Trade school	8.1
College/university	24.7
Smoking status (%)	
Current	29.1
Former	21.0
Never	49.8

**Table 2** displays the reliability for 13 community-level measurement scales derived from the EPOCH 2 instrument overall and separately for urban and rural communities. Table 2 also presents the number of items in each scale and the community-level VPC calculated treating countries as fixed or random effects. Reliabilities have been calculated based on 28 respondents per community, which was the mean number in our sample. Given the number of scale items and respondents per community, relatively high reliabilities were achieved for community-level measures. Observed reliabilities for all scales were >0.8 overall, and in urban/rural analyses and varied from 0.81 for community social cohesion in urban communities to 0.96 for knowledge of the health effects of smoking in rural communities.

**Table 2:** Reliability estimates and variance partitioning coefficients (VPC) for thirteen scales measuring characteristics of the community environment potentially related to cardiovascular disease (CVD).

		Overall			Urban			Rural		
Scale	Numberof items	Reliability	VPC(Rand)	VPC (Fixed)	Reliability	VPC(Rand)	VPC (Fixed)	Reliability	VPC (Rand)	VPC (Fixed)
Community smoking restrictions	5	0.86	0.05	0.05	0.89	0.05	0.06	0.82	0.04	0.04
Smoking restriction preferences	5	0.89	0.09	0.11	0.90	0.11	0.12	0.89	0.09	0.11
Tobacco advertising	7	0.85	0.04	0.05	0.82	0.03	0.04	0.91	0.06	0.09
Promotion of Smoking cessation	3	0.88	0.08	0.10	0.89	0.12	0.14	0.86	0.05	0.07
Social disapproval of smoking	4	0.89	0.06	0.14	0.83	0.06	0.09	0.92	0.06	0.20
Awareness of tobacco legislation	5	0.91	0.08	0.12	0.92	0.09	0.13	0.87	0.05	0.10
Knowledge of health effects of smoking	8	0.93	0.05	0.06	0.89	0.03	0.04	0.96	0.10	0.13
Junk food advertising	5	0.89	0.07	0.10	0.87	0.07	0.09	0.92	0.06	0.11
Fruit & vegetable advertising	5	0.92	0.09	0.10	0.90	0.08	0.09	0.95	0.10	0.12
Promotion of healthydiet	3	0.91	0.07	0.10	0.93	0.10	0.14	0.82	0.03	0.05
Knowledge of dietary causes of CVD	9	0.93	0.07	0.09	0.92	0.08	0.10	0.88	0.03	0.03
Awareness of Foodpolicy legislation	2	0.93	0.12	0.20	0.91	0.09	0.16	0.93	0.16	0.20
Community social cohesion	2	0.86	0.09	0.11	0.81	0.07	0.08	0.92	0.17	0.18

Scales were derived from individual responses to questionnaire items in the EPOCH 2 survey done in 84 urban and rural communities in 5 countries.

Models adjusted for respondent age, sex, level of education and smoking status (current, former, or ever). Overall model additionally adjusts for urban/rural location as a community-specific covariate.

VPC, variance partitioning coefficient; Rand, random effects; Fixed, fixed effects.

Table 2 presents community-level VPCs calculated from separate models which treated countries as either a random or fixed effect. In models where countries were treated as a random effect, VPCs varied from 0.03 for knowledge of health effects of smoking in urban communities and for knowledge of dietary causes of CVD in rural areas to 0.17 for community social cohesion in rural communities. After accounting for the item inconsistency, the between-individual differentials in agreement, and between-country variation, between 3% and 17% of the total variance across the measures of the community environment was at the level of communities, indicating some correlation in response patterns between individuals from the same community.

When countries were treated as a fixed effect in the multilevel models, the resulting VPCs were generally higher and varied from 0.04 for tobacco advertising in urban communities to 0.20 for awareness of food policy legislation overall and in rural communities. The increase in VPC was likely the result of the removal of the separate contribution of country to the total variance when countries were treated as a fixed effect. When countries were treated as a random effect, estimates of the variance parameters at the country level were less statistically reliable due to the limited sample of 5 units at this level.

**Figure 1:**
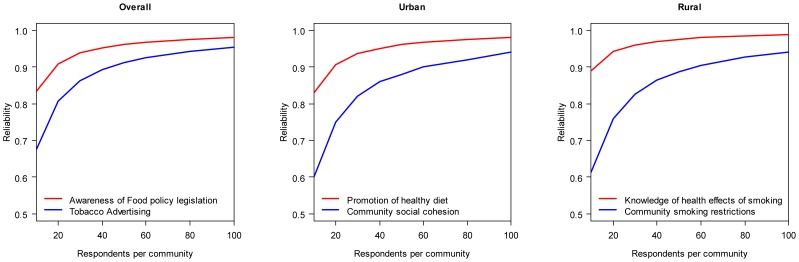
Association between sample size of respondents per community and reliabilities of community-level measures derived from EPOCH 2, overall and in urban and rural communities. In each panel, the measures with the lowest and highest levels of interrater agreement are plotted; all other measures will lie between these two curves.

As noted above, a key question is the optimal sample size per community to achieve reliable results. The association between the sample size of respondents per community and reliability is plotted in **Figure 1** for the scales with the highest and lowest reliabilities overall, and in urban and rural communities. Curves for the other scales not plotted would lie between the two bounds. Based on these findings, a sample of 20 respondents per community would produce a reliability of between 0.75 and 0.94, while a reliabilities based on a sample of 40 respondents would be between 0.86 and 0.97 and a sample of 80 respondents would produce reliabilities between 0.92 and 0.99. Thus, only minimal increases in reliability are achieved by increasing the number of respondents per community beyond 40.

Following reliability analyses, we conducted additional analyses to assess the properties of the 13 scales measuring characteristics of the community potentially related to CVD. First, although each scale was developed to measure different dimensions of the community environment, there is likely some interdependence between scales. **Table 3** presents a correlation matrix of scales measuring the community environment. Correlation analyses indicated positive correlations across scales within identified domains (e.g. community tobacco and nutrition environments) which could feasibly be grouped into a common domain scale in future analyses.

**Table 3:** Correlation matrix of scales measuring characteristics of the community environment.

Scale	Scale 1	Scale 2	Scale 3	Scale 4	Scale 5	Scale 6	Scale 7	Scale 8	Scale 9	Scale 10	Scale 11	Scale 12	Scale 13
1. Community smoking restrictions	1.000												
2. Smoking restriction preferences	0.336	1.000											
3. Tobacco advertising	−0.046	−0.182	1.000										
4. Promotionof Smoking cessation	0.090	0.002	0.112	1.000									
5. Social disapprovalof smoking	−0.183	0.381	−0.183	0.016	1.000								
6. Awarenessof tobacco legislation	0.243	0.206	0.147	0.451	−0.080	1.000							
7. Knowledgeof healtheffects ofsmoking	0.073	0.170	−0.240	0.311	0.070	0.150	1.000						
8. Junk food advertising	0.232	−0.201	0.443	0.533	−0.273	0.339	−0.074	1.000					
9. Fruit & vegetable advertising	0.378	0.026	0.369	0.464	−0.183	0.517	−0.158	0.604	1.000				
10. Promotionof healthy diet	0.339	0.087	0.213	0.766	−0.094	0.496	0.182	0.563	0.729	1.000			
11. Knowledgeof dietary causes of CVD	−0.093	0.148	−0.178	0.402	0.235	0.179	0.470	0.090	−0.013	0.200	1.000		
12. Awarenessof Food policy legislation	0.208	0.375	−0.363	0.282	0.286	0.383	0.445	−0.063	0.073	0.266	0.250	1.000	
13. Community social cohesion	−0.119	−0.121	0.279	0.201	0.044	0.180	0.156	0.227	0.083	0.106	−0.033	0.127	1.000

Second, we undertook multilevel CFA for each of the 13 scales (**Table S1**). These results demonstrated positive factor loadings at the community level for each of the items included in the final scales. This further supports our interpretation that questionnaire items in each scale are capturing a common latent factor at the community level.

## Discussion

In this study, we have two salient findings. First, our results demonstrate that measures of the community tobacco, nutrition, and social environments can be derived reliably in a range of settings using a simple survey and a modest number of respondents. Second, the multilevel CFA supported our interpretation that across the thirteen scales, items were consistent in capturing common latent factors which represent the identified characteristics of the community environment.

Community-level measurement scales in EPOCH are composed of multiple items, thus reducing measurement error that may arise through differences in individual perceptions. This in turn helps reduce the number of respondents needed to achieve a given reliability. The optimal number of respondents observed in this study was about 30 per community (yielding reliabilities of between 0.86 to 0.93) and it would be feasible to recruit a similar number in surveys or epidemiological studies. Our findings that the reliability was consistent across urban and rural areas suggest that the EPOCH instrument may be suitable for use in a wide range of settings.

The individual scales developed in this study have high reliability at the community-level and appear to measure our hypothesized dimensions of the community tobacco, nutrition, and social environments. Subsequent analyses will involve the assessment of the relationship between each of the 13 community-level scales and individual CVD/risk factors using additional data from the PURE study. Within each domain, community-level characteristics that are found to be associated with outcomes of interest will be combined in a single scale representing that domain to avoid potential issues of collinearity which may arise when including multiple community-level characteristics within a single model.

There are two important caveats to our findings. First, the EPOCH study was conducted on a convenience sample of individuals and communities. Survey respondents were drawn from a larger study and likely share certain characteristics (most obviously the willingness to participate in surveys). Such characteristics may contribute to increased intersubjective agreement among EPOCH respondents. Therefore, it seems reasonable to exercise caution and target an overall average of 30–40 individuals per community in to ensure that the reliabilities reported here can be achieved in other settings. Communities with few respondents can, however, contribute important data to the multilevel model provided that the mean number of respondents across all communities is at least 30. Although estimates from communities with fewer observations will tend to be less reliable, they provide partial information which is pooled with the larger communities and contributes to the estimation of the coefficients and variance parameters. [21] Second, in multilevel CFA, an issue arises in the choice of model to use. In the present study, we have chosen a model with a set of loadings and a single factor at both the level of individuals and communities. Although other model choices are possible, we feel that the results of the initial models presented here, which have determined the values of the factor loadings, provide an important initial assessment of the measurement scales in EPOCH and act as a starting point for future research. Further work on the measurement model of EPOCH using a complete multilevel CFA and a rigorous assessment of model fit is required.

In this study, we made no attempt to determine the number of communities needed to support a multilevel study of community influences on health. It is important, however, that such a study have adequate numbers of individuals and communities to achieve a reasonable amount of power. [16] An additional potential limitation is that variance parameters in multilevel models are in different scales depending on the response and type of model (e.g. logit scale for logistic models and normal metric for linear models) and thus not directly comparable. In our analyses, however, we have dealt with this limitation by comparing across models using the reliability statistic and VPC. Both reliability and VPC are scaled between 0 and 1 and thus are comparable across different response types.

Given recent interest in community influences on health, improved measures of ecological settings are needed. The ecometric approach is an important step towards this end as it outlines an appropriate statistical methodology to evaluate such measures. Going forward, the EPOCH instrument is unique in that through its administration within the context of a prospective cohort study, it has the potential to be used in multilevel studies explore changing relationships between measures of the community environment and individual risk factors for CVD.

## Supporting Information

Table S1 **Community-level item-factor loadings from the multilevel factor analysis models for thirteen scales measuring characteristics of the community environment potentially related to cardiovascular disease (CVD) derived from individual responses to questionnaire items in the EPOCH 2 survey done in 84 urban and rural communities in 5 countries.**
(PDF)

Appendix S1 **EPOCH 2 instrument: version September 4, 2008.**
(PDF)
