# Supplementary material for: Environmental Profile of a Community’s Health (EPOCH): An Ecometric Assessment of Measures of the Community Environment Based on Individual Perception
Source: PLoS One. 2012 Sep 4;7(9):e44410. doi: 10.1371/journal.pone.0044410 (PMC3433440; doi:10.1371/journal.pone.0044410)
Supplement: Table S1 — Community-level item-factor loadings from the multilevel factor analysis models for thirteen scales measuring characteristics of the community environment potentially related to cardiovascular disease (CVD) derived from individual responses to questionnaire items in the EPOCH 2 survey done in 84 urban and rural communities in 5 countries. (PDF) [file pone.0044410.s001.pdf]

SUPPLEMENTAL MATERIAL

Environmental Profile of a Community's Health (EPOCH): An ecometric assessment of measures of the community environment based on individual perception

Table S1 Community-level item-factor loadings from the multilevel factor analysis models for thirteen scales measuring characteristics of the community environment potentially related to cardiovascular disease (CVD) derived from individual responses to questionnaire items in the EPOCH 2 survey done in 84 urban and rural communities in 5 countries

	**Item factor loadings**							
**Scale**	2	3	4	5	6	7	8	9
Community smoking restrictions	3.43	3.10	0.23	1.06	-	-	-	-
Smoking restriction preferences	1.75	1.44	0.85	1.14	-	-	-	-
Tobacco advertising	2.16	1.52	2.26	1.56	1.70	1.39	-	-
Promotion of Smoking cessation	1.30	0.91	-	-	-	-	-	-
Social disapproval of smoking	1.93	2.25	1.86	-	-	-	-	-
Awareness of tobacco legislation	1.18	1.25	1.09	0.90		-	-	-
Knowledge of health effects of smoking	4.91	3.43	6.98	7.77	6.41	0.24	0.59	-
Junk food advertising	0.68	1.13	0.91	0.61	-	-	-	-
Fruit & vegetable advertising	2.53	2.54	1.38	1.11	-	-	-	-

Promotion of healthy diet	1.46	1.13	-	-	-	-	-	-
Knowledge of dietary causes of CVD	0.41	7.69	12.69	14.11	4.82	2.08	1.32	7.25
Awareness of Food policy legislation	0.95	-	-	-	-	-	-	-
Community social cohesion	2.44	-	-	-	-	-	-	-

In the multilevel factor analysis model, the loading of the first item has been constrained to 1 in order to make the model identifiable.
